# Intrinsic chemoresistance to gemcitabine is associated with constitutive and laminin-induced phosphorylation of FAK in pancreatic cancer cell lines

**DOI:** 10.1186/1476-4598-8-125

**Published:** 2009-12-21

**Authors:** Wu Huanwen, Liang Zhiyong, Shi Xiaohua, Ren Xinyu, Wang Kai, Liu Tonghua

**Affiliations:** 1 Department of Pathology, Peking Union Medical College Hospital, Chinese Academy of Medical Science, Beijing 100730, PR China

## Abstract

**Background:**

One of the major reasons for poor prognosis of pancreatic cancer is its high resistance to currently available chemotherapeutic agents. In recent years, focal adhesion kinase (FAK), a central molecule in extracellular matrix (ECM)/integrin-mediated signaling, has been thought to be a key determinant of chemoresistance in cancer cells. In this study, we aimed to determine the roles of FAK phosphorylation in the intrinsic chemoresistance of pancreatic cancer cell lines.

**Results:**

Our results showed that, the level of constitutive phosphorylation of FAK at Tyr397 correlated with the extent of intrinsic resistance to Gemcitabine (Gem) in four pancreatic cancer cell lines. Moreover, in Panc-1 cells, which had high expression of pFAK, specific inhibition of constitutive FAK phosphorylation by either RNAi or FRNK overexpression decreased the phosphorylation of Akt, reduced the levels of survivin expression and Bad phosphorylation at Ser136 and increased Gem-induced cytotoxicity and apoptosis. However, in AsPC-1 cells with a low level of pFAK, neither FAK RNAi nor FRNK overexpression affected Gem-induced cell apoptosis. We further found that laminin (LN) induced FAK and Akt phosphorylation in a time-dependent manner, increased the levels of survivin and pBad (pS136) and decreased Gem-induced cytotoxicity and apoptosis in AsPC-1 cells; Specific inhibition of LN-induced FAK phosphorylation by either FAK RNAi or FRNK overexpression suppressed the effects of LN on AsPC-1 cells. Moreover, inhibition of constitutive FAK phosphorylation in Panc-1 cells and LN-induced FAK phosphorylation in AsPC-1 cells by a novel and more specific FAK phosphorylation inhibitor PF-573,228 showed similar results with those of FAK phosphorylation inhibition by FAK RNAi or FRNK overexpression.

**Conclusions:**

In conclusion, our research demonstrates for the first time that both constitutive and LN-induced FAK phosphorylation contribute to increased intrinsic chemoresistance to Gem in pancreatic cancer cell lines and these effects are partly due to the regulation of Akt and Bad phosphorylation and survivin expression. Development of selective FAK phosphorylation inhibitors may be a promising way to enhance chemosensitivity in pancreatic cancer.

## Background

Pancreatic cancer is difficult to treat and patients have an overall 5-year survival rate of <5% and a median overall survival of <6 months [1,2]. Many tumors are already unresectable at diagnosis due to metastasis or the presence of locally advanced disease, and thus the majority of patients are potential candidates for palliative treatment including chemotherapy [3]. Gemcitabine (Gem) is currently the first line drug in the treatment of advanced pancreatic cancer [4,5]. However, due to high intrinsic resistance of pancreatic cancer to currently available agents, clinical trials have shown that Gem alone and Gem-based combination chemotherapy are not likely to achieve great success [3,4,6]. Therefore, new therapeutic strategies are urgently needed. In pancreatic cancer, a combination of conventional chemotherapies with new therapies directly targeted against the molecular changes in pancreatic cancer seems to be the most promising strategy so far [7-9]. Tyrosine kinases have demonstrated great promise as therapeutic targets for cancers, and combinations of appropriate tyrosine kinase inhibitors (TKIs) with cytotoxic agents such as Gem have been demonstrated to improve the prognosis of pancreatic cancer [7,10,11]. Non-receptor tyrosine kinase focal adhesion kinase (FAK) has been shown to be closely related to cancers. FAK expression and (or) phosphorylation was elevated in a variety of cancers and frequently correlated with malignant or metastatic disease and poor patient prognosis [12,13]. Moreover, the modulation of FAK expression and (or) phosphorylation influences the sensitivity of tumor cells to various chemotherapeutic agents, and combination of the selective FAK inhibitors with cytotoxic agents might be a very promising anti-cancer therapy [14-16]. High FAK protein expression is also present in pancreatic cancer, but not significantly related to clinicopathological factors such as tumor histological grade, lymph node metastasis, distant metastasis, histological stage, and overall survival in pancreatic cancer patients [17]. Besides the regulation of FAK expression, another well-understood mode of FAK regulation in cancer cells is phosphorylation, particularly tyrosine phosphorylation [18]. In this study, we first investigated the correlation between the level of constitutive FAK expression and phosphorylation and the extent of chemoresistance in four pancreatic cancer cell lines.

As we know, RNAi downregulates protein expression and thus activity. However, FAK related non-kinase (FRNK) can compete with FAK for focal adhesion binding sites and thus specifically inhibit FAK phosphorylation and downstream signaling without changing expression [19-21]. In our study, we used the two kinds of plasmids (FAK RNAi plasmid and FRNK overexpression plasmid) to further dissect the role of constitutive FAK phosphorylation in the chemoresistance of pancreatic cancer cells that had high level of pFAK.

Recently, a novel small molecule inhibitor, PF-573,228 (here after referred to as PF-228), has been developed to block FAK phosphorylation on Tyr397 and target FAK catalytic activity, which provides an appropriate tool to dissect the role of FAK phosphorylation [22]. Compared with FRNK overexpression, PF-228 is a more specific method to decrease FAK phosphorylation. Therefore, PF-228 was used in our study to confirm the role of FAK phosphorylation in the chemoresistance of pancreatic cancer cells.

FAK is a key molecule in signal transduction from extracellular matrix (ECM) to cells, and it has been reported in recent years that the intrinsic chemoresistance of tumor cells could be induced by ECM-integrin interactions, named cell adhesion-mediated drug resistance (CAM-DR) [23]. Laminin (LN) has been confirmed to be one of the most effective ECM proteins to induce CAM-DR [24-26]. Thus we further explored the role of LN on FAK phosphorylation and the intrinsic chemoresistance in the pancreatic cell line with low level of constitutive FAK phosphorylation.

## Methods

### Antibodies and reagents

Rabbit polyclonal antibodies to pERK1/2, ERK1/2, pAkt(pS473), AKT, pBad(pS112), pBad(pS136) and Bad were from Cell Signaling Technology (Beverly, MA, USA). Mouse monoclonal antibody (mAb) to pFAK (pY397) was purchased from BD Biosciences PharMingen (San Diego, CA, USA). FAK and FRNK (the carboxyl terminus of FAK) proteins, were detected by mAb raised against amino acids 903-1052 of human-origin FAK (Santa Cruz Biotechnology, Santa Cruz, CA, USA). Anti-β-actin, anti-Bcl-2, anti-Bax, anti-survivin, anti-caspase-3 primary antibodies and HRP-conjugated secondary antibodies were all purchased from Santa Cruz.

Gem was purchased from Eli Lilly (Indianapolis, IN, USA). 5-Fluorouracil (5-FU), MTT, insulin, transferrin, selenium, BSA and LN were all supplied by Sigma-Aldrich Chemical (Poole, UK). The FAK inhibitor PF-573,228 was purchased from Tocris (Bristol, UK).

### Cell culture, transfection and generation of stable clones

Pancreatic cancer cell lines were all purchased from ATCC (Rockville, MD, USA). AsPC-1, Panc-1 and BxPC-3 were grown in RPMI 1640 (Invitrogen, Carlsbad, CA, USA) supplemented with 10% (v/v) heat-inactivated fetal calf serum (Gemini Bio-Products, Woodland, CA, USA), whereas MiaPaCa-2 cells were grown in DMEM. All cells were maintained at 37°C in a humidified atmosphere with 5% CO_2_. Cell viability was routinely checked after passage by trypan blue exclusion and was consistently >95%. In all experiments with Gem or 5-FU, cells were allowed to settle for 6 h prior to treatment.

Linearized pcDNA 6.2-GW/EmGFP-miR vector (Invitrogen) which enables increasing knockdown of a single target gene with one construct was used for vector-based RNAi interference (RNAi) analysis. This vector can express microRNA for RNAi analysis in most mammalian cells using the human cytomegalovirus immediate early promoter. Criteria for the selection of the target sequence were as we described previously [27]. Plasmid construction was performed following the manufacturer's instructions. The RNAi vectors (FAK RNAi1, FAK RNAi2) were generated by ligating the annealed DNA oligos into the linearized vector and used to inhibit human FAK gene (GenBank: NM_153831.2). The control vector pcDNA 6.2-GW/EmGFP-miR-neg encodes an mRNA not to target any known vertebrate gene. The annealed oligos in FAK RNAi1 plasmid were:

5'TGCTGAGAAATTTCTCTCTCACGCTGGTTTTGGCCACTGACTGACCAGCGTGAGAGAAATTTCT3' (top strand)

5'CCTGAGAAATTTCTCTCACGCTGGTCAGTCAGTGGCCAAAACCAGCGTGAGAGAGAAATTTCTC3' (bottom strand).

The annealed oligos in FAK RNAi2 plasmid were:

5'TGCTGTTCACCTTCTTTCTGAGGTCTGTTTTGGCCACTGACTGACAGACCTCAAAGAAGGTGAA 3' (top strand)

5'CCTGTTCACCTTCTTTGAGGTCTGTCAGTCAGTGGCCAAAACAGACCTCAGAAAGAAGGTGAAC 3' (bottom strand).

FRNK was PCR amplified from the pRKvsv-FRNK plasmid that was kindly provided by Dr. Kenneth M. Yamada (National Institutes of Health, Bethesda, MD, USA) using the following forward and reverse primers:

5'TCCGGATCCATGGAATCCAGAAGACAGGCTAC 3' and 5'CCGGAATTCTCAGTGTGGCCGTGTCTGCCCTA3'. The PCR products were digested with BamHI and EcoRI and cloned into pcDNA3.1 to generate pcDNA3.1-FRNK plasmid. Empty pcDNA3.1 plasmid was used as control.

Cells were transiently transfected using Lipofectamine2000 reagent (Invitrogen) as suggested by the manufacturer. Stable clones were selected for blasticidin (pcDNA 6.2-GW/EmGFP-miR) or G418 (pcDNA3.1) resistance using standard protocols [27]. Pools of four individual clones were used to avoid artifacts. Parental cells and pools transfected with vector plasmids were used as controls. G418 or blasticidin was removed from the culture media 24 h before functional assays.

### Culture of cells on LN

Cell culture plastics were coated with LN (10 μg/cm^2^) for 2 h at 37°C. LN-coated dishes were rinsed three times with PBS. In all experiments using LN, cells were serum starved for 24 h before the experiments were performed. Cells were then distributed onto LN-coated (LN) or control (plastic) wells and cultured in SITA medium (RPMI 1640 supplemented with 30 nM selenium, 5 μg/ml insulin, 10 μg/ml transferrin, 0.25% (w/v) BSA, 100 U/ml penicillin and 100 μg/ml streptomycin).

### Western blotting

Cells were treated as specified and then lysated in RIPA buffer (Pierce Biotechnology, Rockford, IL, USA) with protease inhibitor mixture tablets and phosphatase inhibitor mixture tablets PhosSTOP (Roche Applied Science, Mannheim, Germany). Protein concentration was determined by the BCA assay (Pierce). The whole-cell lysates were heat denatured at 100°C for 10 min before being run on 8-12% gradient SDS-PAGE. After SDS-PAGE, the proteins were electrotransferred onto nitrocellulosemembranes, blotted with each primary antibody, incubated in secondary antibody and then detected with enhanced chemiluminescence reagent (Amersham Pharmacia Biotech, Bucks, UK) and BioMax MR-1 radiographic film (Kodak, Xiamen, China). Semi-quantitative analysis of band intensities was performed by densitometry using image analysis software Image Pro-Plus (Media Cybernetics, Silver Spring, MD, USA).

### Immunofluorescence

Cells were grown on glass coverslips and fixed with 4% paraformaldehyde for 20 min at room temperature. Fixed cells were then incubated with the primary anti- pFAK (pY397) antibodies overnight, washed with PBS, and incubated again with secondary antibodies conjugated with FITC (green) for 1 h at room temperature. Hoechst 33342 was used to stain the nuclei (blue). Cells incubated with secondary antibodies alone were used as controls. The coverslips were mounted onto slides and cells were viewed by a Leica TCS-SP2 confocal scanning microscope (Leica Microsystems Heidelberg GmbH, Germany).

### Cell viability assay

Cell viability was determined by MTT assay. Logarithmically growing cells were plated at 5 × 103 per well in 96-well plates and allowed to adhere for 6 h. The cells were then cultured in the absence or presence of different concentrations of 5-FU or Gem for the indicated time as specified in the Results. After treatment, 10 μL of the MTT was added to each well to assess the cell viability, and after 4 h at 37°C, the purple-blue MTT formazan precipitate was dissolved in 100 μl of DMSO, and the optical density was measured at 570 nm with a Vmax microplated spectrophotometer (Molecular Devices, Sunnyvale, CA). Each experiment was repeated at least thrice in quadruplicate. The concentration of Gem required to inhibit cell proliferation by 50% (IC50) was calculated using Microsoft Excel software for semi-log curve fitting with regression analysis.

### Clonogenic assay

Colony formation was evaluated using a soft agar clonogenic-forming assay. A volume of 0.5 ml of RPMI1640 containing 10% fetal bovine serum and 0.5% agar was plated on the bottom of 24-well plates. The plates were stored at 4°C to allow the agar to freeze. Cells were treated as specified in the Results, mixed with RPMI1640 containing 10% fetal bovine serum and 0.35% agar and plated onto the 24-well plates that were prepared earlier at 500 cells per well (three wells per group). The plates were then transferred to 37°C. After 14-18 days, colonies were manually counted using a microscope and also visualized by MTT stain.

### Analysis of apoptosis by nuclear morphology

Apoptosis was judged by nuclear condensation. Distilled slides were placed onto the surface of 6-well plates, and then coated or not with LN as described above. Cells were seeded onto the slides, allowed to settle for 6 h and then treated with or without Gem for the indicated time. After treatment, slides were washed with PBS, and cells were fixed with 4% polyformaldehyde for 10 min. The slides were washed again with PBS, and 0.1 ml of Hoechst 33342 at a concentration of 2 μg/ml was added to each slide and incubated in the dark at room temperature for 15 min. The slides were washed three times with PBS, and the cells were examined using a Motic fluorescence microscope and photographed.

### Flow cytometric assay of apoptosis

Phosphatidylserine externalization was analyzed with Annexin-V-FITC/PI kit (BD) by a FACSCalibur flow cytometer (BD) for cell apoptosis according to the manufacturer's instructions.

### Statistical analysis

Results were expressed as the mean ± SE, and statistical differences between groups in these assays were calculated using a Student's two-tailed t test. Significance was defined as P < 0.05 using a two-sided analysis.

## Results

### The level of constitutive phosphorylation of FAK at Tyr397 correlates with the extent of intrinsic chemoresistance to Gem in pancreatic cancer cell lines

**Figure 1:**
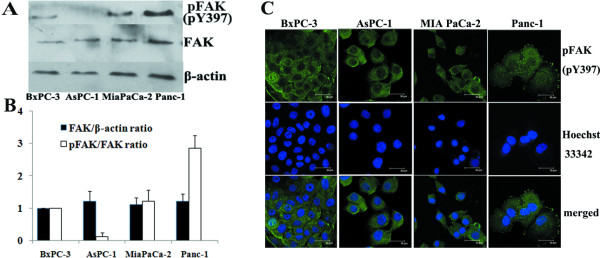
**Constitutive phosphorylation of FAK at Tyr397 in pancreatic cancer cells**. A, Western blot analysis was performed to determine the constitutive level of total FAK and pFAK (pY397) expression in BxPC-3, AsPC-1, MiaPaCa-2 and Panc-1 cells. Equal protein loading was confirmed by β-actin. This figure shows a representative blot of an experiment performed in triplicate. B, For semiquantitation, the intensities of FAK, pFAK and β-actin bands for each cell line were calculated using a densitometer. Bars are mean ± SE of relative FAK/β-actin or pFAK/FAK ratio (normalized to that for BxPC-3 cells, which was given a value of 1) for three independent experiments. C, Confocal microscopy images of the four pancreatic cancer cell lines showing constitutive expression of pFAK (pY397; green) to evaluate FAK phosphorylation status. The same fields stained with Hoechst 33342 (blue) to indicate the position of nuclear DNA are shown in the middle panels and the merged images are shown in the bottom panels.

Western blot was used to determine constitutive FAK and pFAK (pY397) expression in four pancreatic cancer cell lines (BxPC-3, AsPC-1, MiaPaCa-2 and Panc-1) (Fig. 1A-B). Comparable protein levels of total FAK were found in these cell lines, whereas different levels of constitutive FAK phosphorylation were detected in these cell lines. Panc-1 displayed a relatively high level of pFAK (pY397), while MiaPaCa-2 and BxPC-3 cells displayed moderate levels. FAK phosphorylation was lowest in AsPC-1 cells. The different levels of constitutive FAK phosphorylation were further supported by confocal microscopy showing specific peripheral staining of pFAK (pY397) at focal adhesion points (Fig. 1C). Specific pFAK (pY397) staining was more obvious in Panc-1 cells than in the other three cell lines, and little specific staining was observed in AsPC-1 cells.

**Figure 2:**
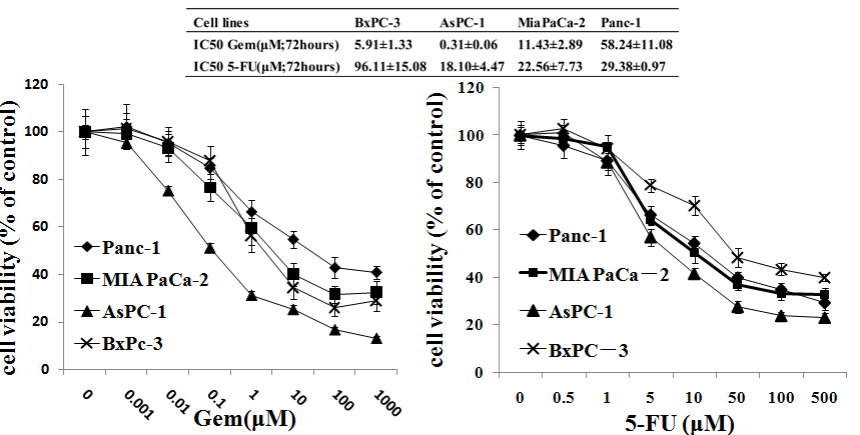
**Intrinsic chemoresistance to Gem and 5-FU in pancreatic cancer cells**. The cell viability of BxPC-3, AsPC-1, MiaPaCa-2 and Panc-1 cells was determined by MTT assay after treatment with increasing doses of Gem or 5-FU for 72 h. And then IC50 was calculated.

MTT assays demonstrated that cells with higher levels of constitutive pFAK (pY397) also showed higher intrinsic chemoresistance to Gem treatment (Fig. 2). The IC50 of Gem for Panc-1 cells (58.24 μM) was approximately 5 times higher than that for MiaPaCa-2 cells (11.43 μM; P < 0.01), one log higher than that for BxPC-3 cells (5.91 μM; P < 0.01) and two logs higher than that for AsPC-1 cells (0.31 μM; P < 0.01). Spearman analysis showed that the IC50 of Gem in these four cell lines significantly correlated with the level of constitutive pFAK (pFAK/FAK ratio) (r = 1; P < 0.05). There was no significant correlation between pFAK level and the IC50 of 5-FU and between total FAK protein level (FAK/β-actin ratio) and the IC50 of Gem or 5-FU.

Taken together, these results suggested that constitutive FAK phosphorylation was positively correlated with the intrinsic chemoresistance to Gem in pancreatic cancer cells.

### Both FAK RNAi and FRNK overexpression decrease the phosphorylation of FAK and Akt in Panc-1 cells

**Figure 3:**
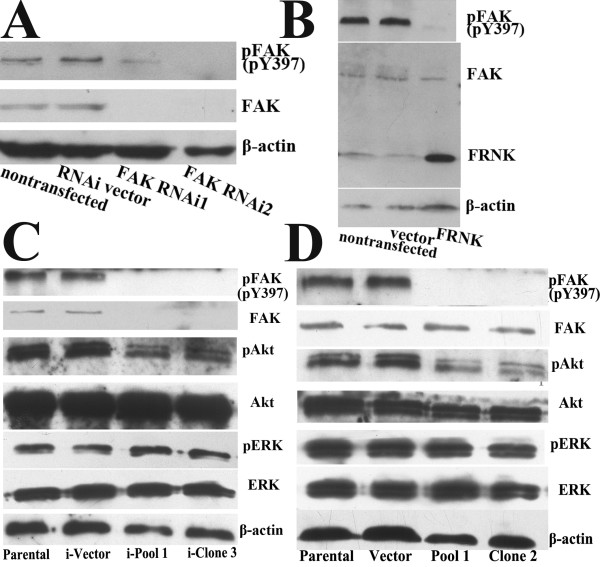
**Effects of FAK RNAi and FRNK overexpression on the expression and phosphorylation of FAK in Panc-1 cells**. Western blot analysis showed expression of FRNK, total FAK and pFAK (pY397) in Panc-1 cells transiently transfected with FAK RNAi plasmids (FAK RNAi1 and FAK RNAi2) for five days (A) or pcDNA3.1-FRNK plasmid (FRNK) for three days (B). Parental cells and their respective vector-transfected (RNAi vector, vector) cells served as the control. C, FAK RNAi2 plasmid-transfected (i-Pool 1, i-Clone 3) and vector-transfected (i-Vector) clones derived from Panc-1 cells were obtained using blasticidin as a selection marker. Western blot showed expression of pFAK (pY397), p-Akt (pS473), p-ERK 1/2 and their total proteins in the cells. D, pcDNA3.1-FRNK plasmid-transfected (Pool 1, Clone 2) and empty vector-transfected (Vector) clones were obtained using G418 as a selection marker. Western blot showed expression of pFAK (pY397), p-Akt (pS473), p-ERK 1/2 and their total proteins in the cells. The membranes were probed with anti-β-actin antibody to ensure even loading of proteins in each lane.

We used two different kinds of plasmids to downregulate FAK phosphorylation in Panc-1 cells, which had higher constitutive pFAK (pY397) level. As expected, transient transfection experiments showed that both methodological approaches could inhibit FAK phosphorylation in Panc-1 cells. Compared with nontransfection and vector-transfection controls, transient transfection of RNAi plasmids (FAK RNAi1 and FAK RNAi2) resulted in downregulation of FAK protein levels and subsequent reduction of pFAK (pY397) levels (Fig. 3A), whereas transfection of pcDNA3.1-FRNK plasmid decreased pFAK levels without changing total FAK expression (Fig. 3B).

Individual clones and pools of Panc-1 cells transfected with FAK RNAi2, pcDNA3.1-FRNK were obtained and examined for total FAK and pFAK (pY397) expression. Results observed in the stable clones were similar to the transient transfection experiments (Fig. 3C-D). Akt and ERK1/2 are two key kinases that are downstream of FAK, and they are important for mediating cell survival. In accord with decreased pFAK (pY397) levels, Panc-1 cells stably transfected with either FAK RNAi2 or pcDNA3.1-FRNK plasmid showed decreased Akt phosphorylation. However, the levels of total Akt, total ERK1/2 and pERK1/2 were not affected. RT-PCR analysis also showed that FAK mRNA level was decreased in Panc-1 cells stably transfected with FAK RNAi2 (data not shown).

These results confirmed that both FAK RNAi and FRNK overexpression decreased the phosphorylation of FAK and downstream kinase Akt in Panc-1 cells. To avoid artifacts resulting from the use of single clones of transfected cells, a pool of four individual clones was used for further experiments.

### Both FAK RNAi and FRNK overexpression enhance Gem-induced cytotoxicity and apoptosis in Panc-1 cells

**Figure 4:**
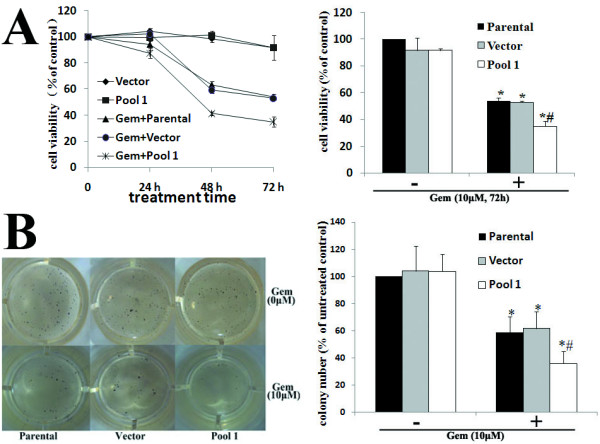
**Effects of FRNK overexpression on Gem-induced chemoresistance in Panc-1 cells**. A, The cell viability of parental Panc-1 cells and empty vector-transfected (Vector) and pcDNA3.1-FRNK plasmid-transfected (Pool 1) cells was determined by cell proliferation assays after treatment with or without 10 μM Gem for 24, 48 and 72 h. Results were expressed as the percentages of viable cells compared with parental cells without Gem treatment (left). The cell viability was statistically compared at 72 h after Gem treatment. Bars represent the mean of three independent experiments ± SE. *, P < 0.05, vs. parental cells without Gem treatment; #, P < 0.05, vs. parental or vector cells with Gem treatment (right). B, Parental Panc-1 cells and vector and pool 1 cells were treated with or without 10 _M Gem for 24 h. Cells were then trypsinized and seeded in equal numbers into 24-well plates for clonogenic assay. After14 to 18 days, the mean number of the colonies was counted (left). The inhibition rate was defined by comparison of the colony number of each group with that of parental cells without Gem treatment. Bars represent the mean of three independent experiments ± SE. *, *P *< 0.05, vs. parental cells without Gem treatment; ^#^, *P *< 0.05, vs. parental or vector cells with Gem treatment (right).

Cytotoxicity was determined by MTT and clonogenic assays. Gem significantly inhibited Panc-1 cell viability in a time-dependent manner (Fig. 4A, left). Stable pool cells overexpressing FRNK had no significant difference in proliferation compared with parental and vector cells. However, pool cells overexpressing FRNK demonstrated an increased sensitivity to Gem treatment. After 72 h of Gem treatment, the viability was approximately 20% lower in pool cells overexpressing FRNK (P < 0.05) (Fig. 4A, right). Similar results were obtained in clonogenic assays (Fig. 4B).

**Figure 5:**
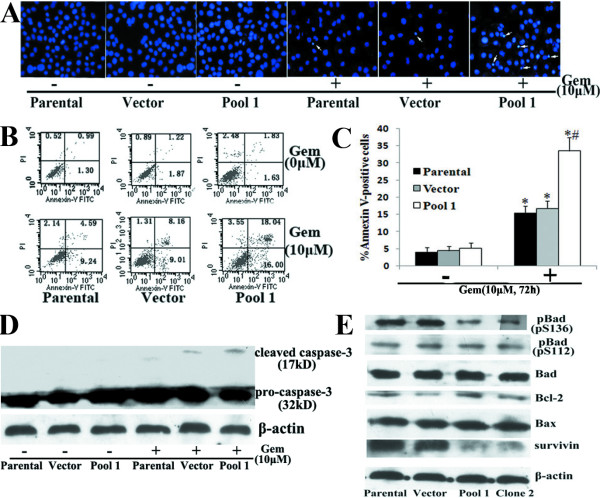
**Effects of FRNK overexpression on Gem-induced apoptosis in Panc-1 cells**. Parental Panc-1 cells and empty vector-transfected (Vector) and pcDNA3.1-FRNK plasmid-transfected (Pool 1) cells were treated with or without 10 μM Gem for 72 h and subjected to cell apoptosis analysis by Hoechst staining (the arrow indicates the apoptotic cells) (A), flow cytometry analysis of Annexin-V labeling (B) and western blot analysis of cleaved caspase-3 protein expression (C). Bars represent the mean of three independent experiments ± SE. *, *P *< 0.05, vs. parental cells without Gem treatment; ^#^, P < 0.05, vs. parental cells or vector cells with Gem treatment. D, Western blot analysis was used to detect the expression of Bad, p-Bad (pS136), p-Bad (pS112), Bcl-2, Bax and survivin in parental cells and vector and pool 1 cells. The membranes were probed with anti-β-actin antibody to ensure even loading of proteins in each lane.

Apoptosis is considered as the major mechanism of chemotherapy-induced cell death [28]. We further determined the effects of FRNK overexpression on Gem-induced apoptosis in Panc-1 cells. Cell apoptosis was analyzed by Hoechst staining of nuclei, Annexin-V analysis of externalized phosphatidylserine and western blot analysis of cleaved caspase-3 protein (Fig. 5A-D). Compared with control groups, pool cells overexpressing FRNK were more sensitive to Gem-induced apoptosis, which was demonstrated by an increased proportion of condensed nuclei, significantly higher of Annexin-V positivity (P < 0.05) and more cleaved caspase-3 protein expression. However, FRNK overexpression did not significantly affect the apoptosis of Panc-1 cells in the absence of Gem. Apoptosis-associated proteins Bax, Bcl-2, BAD and survivin have all been demonstrated to be involved in the chemoresistance of pancreatic cancer cells and be regulated by FAK or Akt [29-33]. Thus, we investigated whether inhibition of FAK activity by FRNK overexpression might modulate these proteins and thereby regulate apoptosis in Panc-1 cells. Compared with parental cells and vector cells, clone 2 and pool 1 cells transfected with pcDNA3.1-FRNK showed a decrease in survivin expression and Bad phosphorylation at Ser136 but did not affect Bax, Bcl-2 or Bad expression or Bad phosphorylation at Ser112 (Fig. 5E). Similar results were obtained in Panc-1 cells stably transfected with the FAK RNAi2 plasmid (data not shown).

These results clearly showed that, inhibition of constitutive FAK phosphorylation was sufficient to render Panc-1 cells more chemosensitive to Gem. It indicated that constitutive pFAK was at least partially responsible for Gem chemoresistance in pancreatic cancer lines and suggested that the mechanisms might be related to survivin expression and pBad (pS136) level.

### LN induces the phosphorylation of FAK and its downstream kinase Akt in AsPC-1 cells

**Figure 6:**
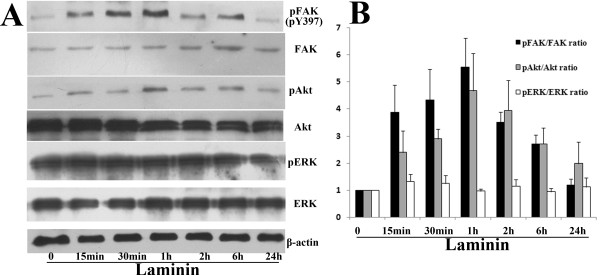
**Effect of LN on FAK phosphorylation and its downstream signaling kinases (Akt and ERK1/2) activity in AsPC-1 cells**. A, AsPC-1 cells were treated as described in results for the indicated time and then subjected to western blot analysis to detect the expression of pFAK (pY397), p-Akt (pS473), p-ERK1/2 and their total proteins. The membranes were probed with anti-β-actin antibody to ensure even loading of proteins in each lane. B, For semiquantitation, the intensities of bands for each cell line were calculated using a densitometer. Bars are mean ± SE of relative pFAK/FAK, pAkt/Akt or pERK/ERK ratio (normalized to that of 0 min, which was given a value of 1) for three independent experiments.

AsPC-1 cells, which had lower level of FAK phosphorylation, were plated on LN for different time in SITA medium. The levels of FAK, Akt and ERK phosphorylation in cells were then examined (Fig. 6A-B). A low level of constitutively activated FAK and Akt was found in AsPC-1 cells, and a rapid and strong stimulation of FAK and Akt phosphorylation was induced by LN. The levels of phosphorylated FAK and Akt began to rise at 15 min and peaked at 1 h after adhesion to LN, followed by a decline over 24 h. In contrast, a significant basal level of phosphorylated ERK was observed in AsPC-1 cells, and no significant change was induced by LN. The levels of total FAK, Akt and ERK protein and pERK in AsPC-1 cells were all not significantly affected by LN.

**Figure 7:**
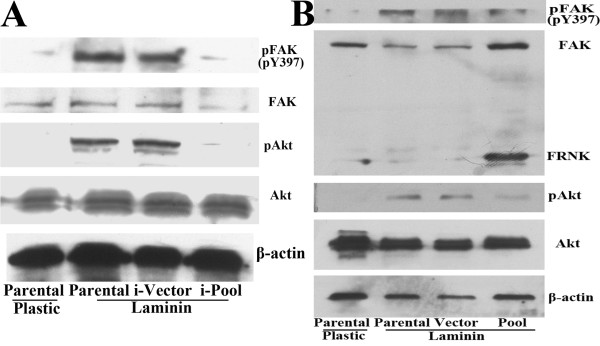
**Effects of FAK RNAi and FRNK overexpression on LN-mediated FAK and Akt activation**. After replating on plastic or LN for 1 h, parental AsPC-1 cells and FAK RNAi2-transfected pool cells (i-Pool) (A), pcDNA3.1-FRNK-transfected pool cells (Pool) (B) and their respective vector-transfected clones (i-Vector, Vector) were collected and used for western blot analysis to detect the expression of pFAK (pY397), p-Akt (pS473) and their total proteins. The membranes were probed with anti-β-actin antibody to ensure even loading of proteins in each lane.

To determine whether LN-induced Akt activation in AsPC-1 cells was dependent on FAK, pool cells transfected with FAK RNAi2, pcDNA3.1-FRNK or their respective vector control were obtained. The effect of LN on Akt activation was almost completely blocked by inhibition of FAK phosphorylation through either FAK RNAi or FRNK overexpression (Fig. 7A-B).

These results indicated that in AsPC-1 cells, LN induced FAK and Akt phosphorylation in a time-dependent manner, and LN-induced Akt phosphorylation was mediated by FAK activation.

### LN suppresses Gem-induced cytotoxicity and apoptosis in AsPC-1 cells

**Figure 8:**
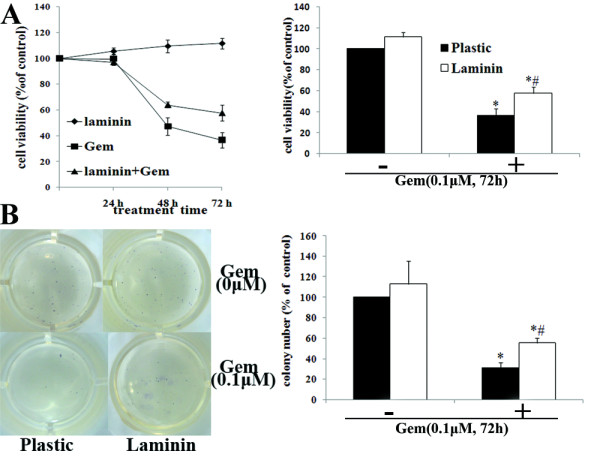
**Effects of LN on Gem-induced cytotoxity in AsPC-1 cells**. AsPC-1 cells were treated as described in results. A, The cell viability of AsPC-1 cells of different groups was determined by MTT assays. Results were expressed as the percentages of viable cells compared with cells on plastic without Gem treatment (left). The cell viability was statistically compared at 72 h after Gem treatment (right). B, The long-term clonogenic potential of pancreatic cancer cells was evaluated by clonogenic assay. AsPC-1 cells on plastic or LN were treated with or without Gem (0.1 μM) for 24 h in SITA medium. Cells were then trypsinized and seeded in equal numbers into 24-well plates. After14 to 18 days, the mean number of the colonies was counted, and the inhibition rate was defined by comparison of the colony number of each group with that of cells on plastic without Gem treatment.

Our results demonstrated that LN protected AsPC-1 cells from Gem-induced cytotoxicity in a time-dependent manner, and the protective effect was most obvious at 72 h after Gem treatment (Fig. 8A). Colony-forming assays confirmed the protective effect of LN on Gem-induced cytotoxicity (Fig. 8B).

**Figure 9:**
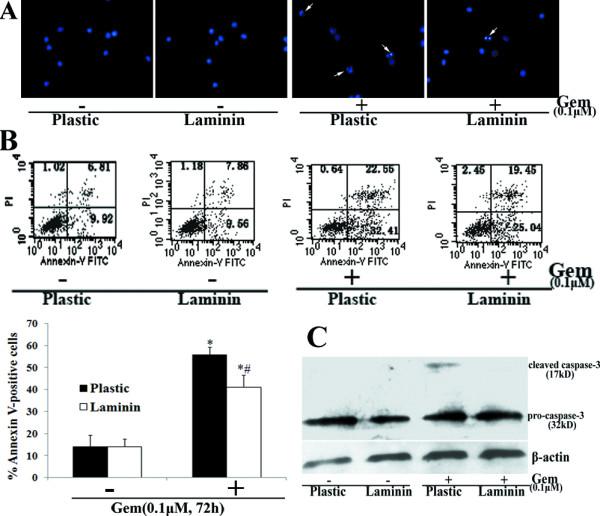
**Effects of LN on Gem-induced apoptosis in AsPC-1 cells**. AsPC-1 cells were treated as described in results. After Gem treatment for 72 h, cell apoptosis was examined by Hoechst staining (the arrow indicates the apoptotic cells) (A), flow cytometry analysis of Annexin-V labeling (B) and western blot analysis of cleaved caspase-3 protein expression (C). Bars represent the mean of three independent experiments ± SE. *, P < 0.05, vs. cells on plastic without Gem treatment; #, P < 0.05, vs. cells on plastic treated with Gem.

Moreover, after Gem treatment, AsPC-1 cells plated on LN demonstrated decreased apoptosis compared with those on plastic (P < 0.05) (Fig. 9A-C). Data also revealed that LN did not significantly protect cells without Gem treatment from apoptosis.

**Figure 10:**
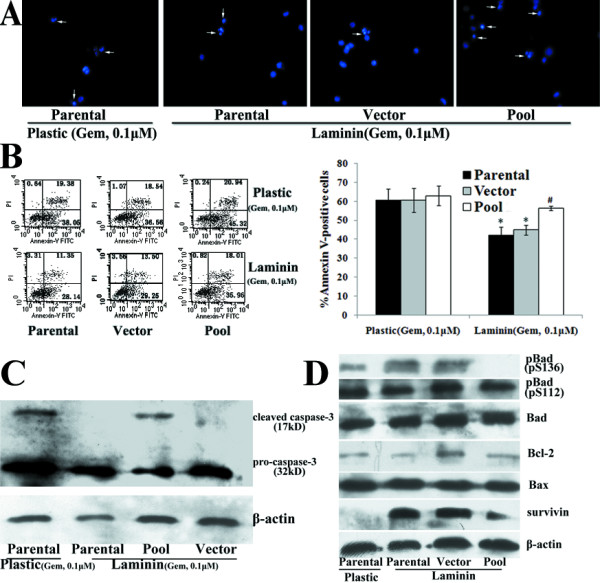
**Effects of FRNK overexpression on LN-mediated Gem chemoresistance**. Parental AsPC-1 cells and vector-transfected (Vector) and pcDNA3.1-FRNK-transfected (Pool) clones were treated with Gem (0.1 μM) for 72 h. Cell apoptosis was examined by Hoechst staining (the arrow indicates the apoptotic cells) (A), flow cytometry analysis of Annexin-V labeling(B) and western blot analysis of cleaved caspase-3 protein expression(C). Bars represent the mean of three independent experiments ± SE. *, P < 0.05, vs. parental cells on plastic; #, P < 0.05, vs. parental cells or vector cells on LN. D, After plated on LN for 24 h, western blot analysis was used to detect the expression of Bad, p-Bad (pS136), p-Bad (pS112), Bcl-2, Bax and survivin in parental AsPC-1 cells and vector and pool clones on LN. Parental cells on plastic were used as control. The membranes were probed with anti-β-actin antibody to ensure even loading of proteins in each lane.

LN also caused an increase in the expression of survivin and the phosphorylation of Bad at Ser136 but did not affect Bax, Bcl-2 or Bad expression or Bad phosphorylation at Ser112 in AsPC-1cells (Fig. 10D).

Collectively, these findings suggested that LN might mediate the intrinsic chemoresistance to Gem in AsPC-1 cells.

### Effects of FAK RNAi and FRNK overexpression on LN-mediated Gem chemoresistance in AsPC-1 cells

When cultured on LN, pool cells expressing FRNK demonstrated a significant increase in Gem-induced apoptosis, compared with parental cells and vector cells (P < 0.05) (Fig. 10A-C). However, FRNK overexpression did not significantly affect Gem-induced apoptosis in AsPC-1 cells on plastic (Fig. 10B). Moreover, inhibition of FAK phosphorylation by FRNK overexpression antagonized the effects of LN on survivin expression and Bad phosphorylation at Ser136 in AsPC-1 cells (Fig. 10D). Similar results were observed with FAK RNAi in AsPC-1 cells (data not shown). These results indicated that in AsPC-1 cells, LN-induced FAK phosphorylation mediated the intrinsic chemoresistance to Gem, and this effect might be related with the regulation of survivin and pBad (pS136) level

### Effects of PF-228 on Gem-induced apoptosis in pancreatic cancer cells

PF-228, a novel FAK inhibitor, has become available recently. It specifically blocks FAK phosphorylation and thus targets FAK catalytic activity. PF-228 is a more specific method to decrease FAK phosphorylation compared with FRNK overexpression. Therefore, in our study PF-228 was further applied to confirm the role of FAK phosphorylation in the chemoresistance of pancreatic cancer cells.

**Figure 11:**
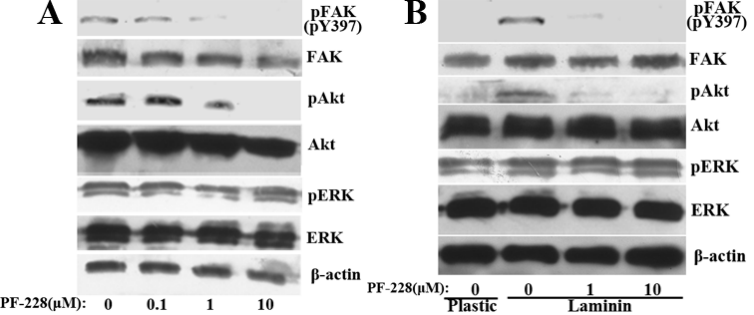
**Effects of PF-228 on the phosphorylation of FAK, Akt and ERK in pancreatic cancer cells**. A, Panc-1 cells were treated with or without the indicated concentrations of PF-228 for 1 h. B, After pretreated with or without the indicated concentrations of PF-228 for 30 min in serum-free medium, suspended AsPC-1 cells were plated onto plastic or LN-coated plates for 1 h in the continued presence or absence of PF-228. Western blot showed expression of pFAK (pY397), p-Akt (pS473), p-ERK 1/2 and their total proteins in the cells. The membranes were probed with anti-β-actin antibody to ensure even loading of proteins in each lane.

We used PF-228 to downregulate constitutive FAK phosphorylation in Panc-1 cells and LN-induced FAK phosphorylation in Aspc-1 cells respectively. PF-228 could inhibit both constitutive and LN-induced FAK phosphorylation in a dose-dependent manner (Fig. 11A-B). 1 μM PF-228 was sufficient to efficiently block both constitutive FAK phosphorylation in Panc-1 cells and LN-induced FAK phosphorylation in Aspc-1 cells. Consistent with the results of FAK phosphorylation inhibition by FAK RNAi and FRNK overexpression, specific inhibition of FAK phosphorylation by PF-228 led to the corresponding inhibition of AKT but not ERK phosphorylation in Panc-1 cells and Aspc-1 cells. The levels of total FAK, Akt and ERK protein were not significantly affected.

**Figure 12:**
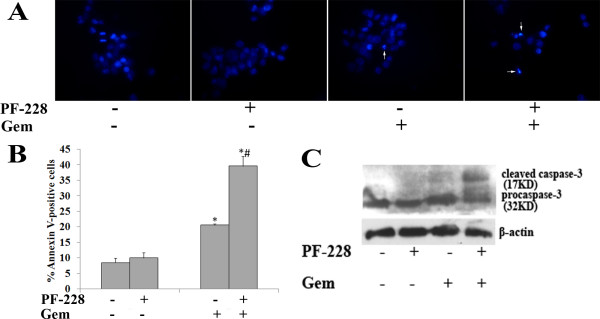
**Effects of PF-228 on Gem-induced apoptosis in Panc-1 cells**. Panc-1 cells were pretreated with or without PF-228 (1 μM) for 1 h and then treated with or without 10 μM Gem for 72 h in the continued presence or absence of PF-228. The cells were subjected to apoptosis analysis by Hoechst staining (the arrow indicates the apoptotic cells) (A), flow cytometry analysis of Annexin-V labeling (B) and western blot analysis of cleaved caspase-3 protein expression (C). Bars represent the mean of three independent experiments ± SE. *, P < 0.05, vs. Panc-1 cells without Gem treatment; #, P < 0.05, vs. Gem-treated Panc-1 cells without PF-228 pretreatment.

**Figure 13:**
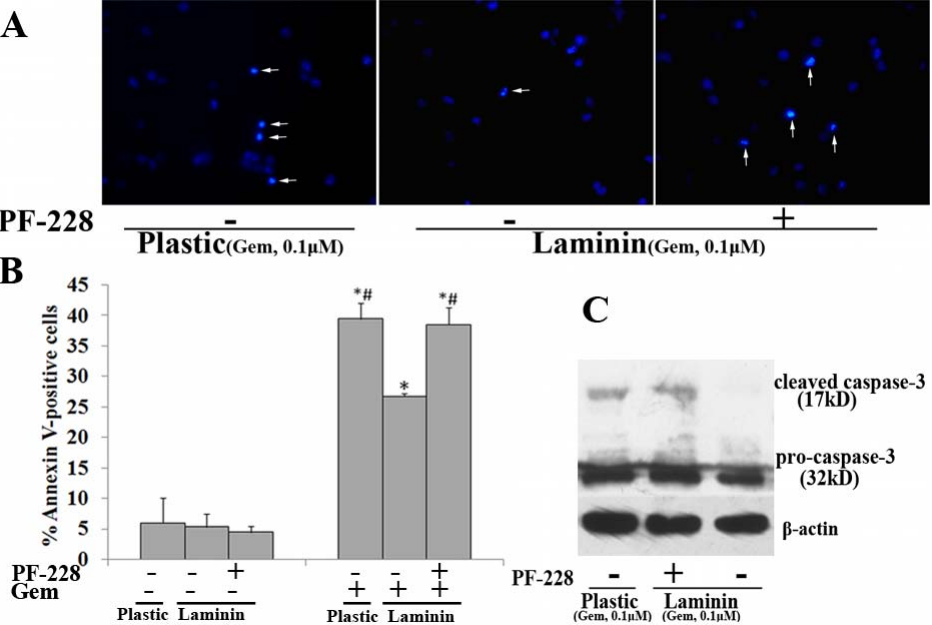
**Effects of PF-228 on LN-mediated Gem chemoresistance in AsPC-1 cells**. After pretreated with or without PF-228 (1 μM) for 30 min in serum-free medium, AsPC-1 cells were plated onto plastic or LN-coated 6-well plates and treated with Gem (0.1 μM) for 72 h in the continued presence or absence of PF-228. Cell apoptosis was examined by Hoechst staining (the arrow indicates the apoptotic cells) (A), flow cytometry analysis of Annexin-V labeling(B) and western blot analysis of cleaved caspase-3 protein expression(C). Bars represent the mean of three independent experiments ± SE. *, P < 0.05, vs. AsPC-1 cells without Gem treatment; #, P < 0.05, vs. Gem-treated AsPC-1 cells without PF-228 pretreatment on LN.

We further determined the effects of PF-228 on Gem-induced apoptosis in pancreatic cancer cells. Cell apoptosis was determined by methods as described above. Consistent with the results of FAK RNAi and FRNK overexpression, PF-228 rendered Panc-1 cells more sensitive to Gem-induced apoptosis (Fig. 12A-C), while in AsPC-1 cells PF-228 treatment antagonized LN-mediated Gem chemoresistance (Fig. 13A-C), which was demonstrated by an increased proportion of condensed nuclei, significantly higher of Annexin-V positivity (P < 0.05) and more cleaved caspase-3 protein expression. However, PF-228 treatment alone did not significantly affect the apoptosis of Panc-1 cells on plastic or Aspc-1cells on LN. (Fig. 12C and 13C)

**Figure 14:**
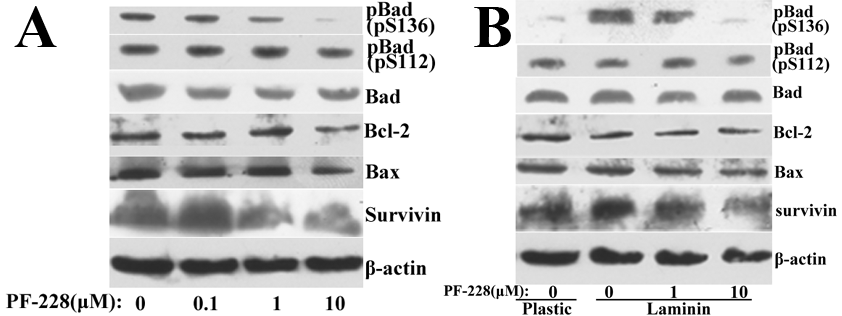
**Effects of PF-228 on the expression of apoptosis-associated proteins in pancreatic cancer cells**. A, Panc-1 cells were treated with or without the indicated concentrations of PF-228 for 24 h. B, After pretreated with or without the indicated concentrations of PF-228 for 30 min in serum-free medium, AsPC-1 cells were plated onto plastic or LN for 24 h in the continued presence or absence of PF-228. Western blot analysis was used to detect the expression of Bad, p-Bad (pS136), p-Bad (pS112), Bcl-2, Bax and survivin. The membranes were probed with anti-β-actin antibody to ensure even loading of proteins in each lane.

Consistent with the results of FAK RNAi and FRNK overexpression, PF-228 decreased survivin expression and Bad phosphorylation at Ser136 in Panc-1 cells and antagonized the effects of LN on survivin expression and Bad phosphorylation at Ser136 in AsPC-1 cells (Fig. 14A-B).

These results further confirmed that, constitutive and LN-induced FAK phosphorylation was at least partially responsible for the intrinsic chemoresistance to Gem in pancreatic cancer cells.

## Discussion

Pancreatic cancer remains a major therapeutic challenge. High resistance to chemotherapy is considered a common phenomenon and one of the major reasons for poor prognosis in pancreatic cancer [2]. Links between tyrosine kinases and tumor chemoresistance have attracted more and more attention in recent years [7]. The combination of targeted therapy against tyrosine kinases and conventional approved drugs such as Gem has proven effective in both preclinical and clinical settings [8,10,11].

A pivotal role of the non-receptor tyrosine kinase FAK has been demonstrated in a variety of human tumors by immunohistochemical and molecular analysis. FAK expression or phosphorylation is elevated in ovarian, breast, head and neck, thyroid, esophageal, colon, liver and pancreatic cancers, indicating that FAK might be a novel therapeutic target and prognostic marker for these malignancies [12,13,17,23]. Consistent with a previous study [17], all four pancreatic cancer cell lines that we tested showed high FAK expression at the protein level. In recent studies, researchers have begun to hypothesize that FAK is a key determinant of chemoresistance since the modulation of FAK function through antisense oligonucleotides or RNAi influences the sensitivity of different kinds of tumor cells to various chemotherapeutic agents [15,16,34]. Herein, we examined whether constitutive FAK protein expression in pancreatic cancer cells correlated with the intrinsic chemoresistance to Gem or 5-FU. However, our study showed total FAK protein expression which was similar among all four cell lines, did not correlate with Gem or 5-FU chemoresistance. It has also been reported previously that FAK protein expression might not be a prognostic marker for pancreatic cancer patients [17]. Tyrosine 397 is the major site of autophosphorylation in FAK. Phosphorylation at Tyr397 correlates with increased catalytic activity of FAK and is important for tyrosine phosphorylation of focal-adhesion-associated proteins [35,36]. Our study here showed that constitutive pFAK (pY397) levels positively correlated with Gem chemoresistance in pancreatic cancer cell lines. This indicates that the phosphorylated active form of FAK may be of greater biological significance compared with the total expression.

We demonstrated herein that specific RNAi against FAK reduced FAK expression, decreased FAK phosphorylation and thus suppressed the intrinsic chemoresistance to Gem in Panc-1 cells, which had a high level of pFAK (pY397). Our results indicate that FAK is a potential target for pancreatic cancer treatment. The C-terminal non-catalytic domain of FAK termed FRNK functions as a competitive inhibitor of FAK and ectopic expression of FRNK specifically inhibits FAK autophosphorylation at Tyr397 and thus attenuates its activity [19,20]. In our study, FRNK overexpression enhanced Gem-induced cytotoxicity and apoptosis to a similar extent as FAK RNAi in Panc-1 cells. However, FRNK overexpression did not significantly affect Gem-induced apoptosis in AsPC-1 cells that had low level of pFAK (pY397). These results demonstrate that constitutive FAK phosphorylation contributes to the intrinsic chemoresistance to Gem in pancreatic cancer cells. Previous study in breast cancer cells has also found that FRNK overexpression inhibited the activation of FAK and PKB and thus enhanced chemotherapy-induced cell apoptosis [37]. Small molecule inhibitors of FAK phosphorylation (such as PF-573,228, PF-562,271, TAE226, 1,2,4,5-Benzenetetraamine tetrahydrochloride) have been developed in recent years [22,38-40]. PF-562,271 is a potent inhibitor of both FAK and the related kinase Pyk2, while TAE226 is an effective inhibitor of both FAK and insulin-like growth factor I receptor [38]. Therefore, a commercially available and more specific inhibitor of FAK phosphorylation, PF-228, was chosen in our study. Compared with FRNK, PF-228 can more specifically block FAK autophosphorylation both in normal and tumor cells. As expected, inhibition of constitutive FAK phosphorylation by PF-228 also decreased the intrinsic chemoresistance to Gem in Panc-1 cells. It further confirms the role of constitutive FAK phosphorylation in the intrinsic chemoresistance to Gem in pancreatic cancer cells and indicates development of selective FAK phosphorylation inhibitors may be a promising way to enhance chemosensitivity in pancreatic cancer. Interestingly, FRNK overexpression or PF-228 alone did not induce apoptosis in pancreatic cancer cells. Consistent with this, a previous study reported that PF-228 had no effect on the growth or apoptosis of normal or cancer cells [22].

In recent years, ECM proteins such as LN, fibronectin and collagen I have been thought to be associated with the intrinsic chemoresistance of many cancers. This phenomenon called CAM-DR represents a novel intrinsic pathway for evading drug-induced apoptosis [23,25,26,41]. Previous data have also shown that α6β1 integrins, major LN-binding receptor, are highly expressed in pancreatic cancer tissues and cell lines, including AsPC-1 [25,42]. Our study demonstrated that LN preventedAsPC-1 cells from Gem-induced cytotoxicity and apoptosis. It indicates that CAM-DR might be an important intrinsic chemoresistance mechanism in pancreatic cancer. Moreover, it has also been reported that Type I collagen reduced apoptosis of AsPC-1 cells in response to 5-FU [43]. FAK functions as a critical intracellular mediator in the ECM-integrin-initiated signaling pathway [44,45]. Our studies found that LN induced FAK phosphorylation in a time-dependent manner in AsPC-1 cells, and FAK phosphorylation inhibition by either RNAi or FRNK overexpression antagonized the effect of LN on Gem chemoresistance. The role of LN-induced FAK phosphorylation in LN-mediated Gem chemoresistance was further confirmed by using the more specific inhibitor of FAK phosphorylation, PF-228. These results indicate that induced FAK phosphorylation is involved in LN-mediated chemoresistance to Gem and further confirm FAK as a promising therapeutic target in pancreatic cancer. Targeted therapy against FAK by methods such as using specific phosphorylation inhibitors could potentially be used to inhibit the cell-ECM interaction and thus suppress CAM-DR.

Akt and ERK are key downstream effectors of FAK in mediating cell survival [44,46,47]. Upon integrin binding to ECM or other stimuli, FAK is autophosphorylated at Tyr397, which provides a high-affinity docking site for several proteins including the p85 subunit of PI3K and the Src kinase. Src can further phosphorylate FAK at several additional sites, including Tyr925. The phosphorylation of Tyr397, as well as of Tyr925, creates a binding site for the Grb2-SOS complex which then permits signaling to the RAS-MAPK cascade [35]. Our research showed that specific inhibition of constitutive FAK phosphorylation decreased Akt but not ERK phosphorylation in Panc-1 cells. Similarly, in Aspc-1 cells, LN-induced FAK phosphorylation was accompanied by Akt but not ERK activation, and specific inhibition of FAK phosphorylation decreased LN-induced Akt activation. These data indicate that Akt might be involved in the intrinsic chemoresistance mediated by FAK phosphorylation. These results are supported by previous reports that the PI-3K-Akt pathway was responsible for Gem chemoresistance in pancreatic cancer in vivo and in vitro. Moreover, PI-3K-Akt has also been shown to be involved in CAM-DR in small cell lung cancer [26,48].

Apoptosis-associated proteins have been reported to relate with chemoresistance in malignant tumors including pancreatic cancers [29-32]. Pro-apoptosis protein Bad is modulated by phosphorylation at two sites, Ser112 (ERK-dependent) and Ser136 (Akt-dependent). Phosphorylation prevents Bad from binding either Bcl-2 or Bcl-XL and thus suppresses apoptosis. Inhibition of phosphorylation at either site might sensitize tumor cells to chemotherapy [31,49]. In our study, corresponding with the alteration of Akt, pBad (pS136) was regulated by constitutive and induced FAK phosphorylation in pancreatic cancer cells. In addition, survivin exression was also regulated by FAK phosphorylation. These data imply that pBad and survivin might contribute to the intrinsic chemoresistance mediated by constitutive and LN-induced FAK phosphorylation.

## Conclusions

Our research demonstrates for the first time that both constitutive and LN-induced phosphorylation of FAK contribute to the intrinsic chemoresistance to Gem in pancreatic cancer cell lines. This effect may be partially due to the regulation of Akt signaling pathway and apoptosis-associated proteins. Our results suggest that FAK can be an attractive therapeutic target for pancreatic cancer, and the development of selective FAK phosphorylation inhibitors may be a promising way to enhance Gem chemosensitivity in pancreatic cancer.

## Competing interests

The authors declare that they have no competing interests.

## Authors' contributions

WH carried out the molecular studies and drafted the manuscript. SX helped to establish the stable clones. RX and WK helped to determine the cell viability by MTT assays. LT and LZ conceived of the study, and participated in its design and coordination and helped to draft the manuscript. All authors read and approved the final manuscript.
